# A retrospective evaluation of acute kidney injury and effects of renal replacement therapy in septic or nonseptic critically ILL patients

**DOI:** 10.1186/2197-425X-3-S1-A265

**Published:** 2015-10-01

**Authors:** İ Başeğmez, FŞ Kahveci, NK Girgin, E Işıktaş, DK Çalışkan, RSİ Aydın, R İşçimen

**Affiliations:** University of Uludag, School of Medicine, Anesthesiology and ICU, Bursa, Turkey; University of Uludag, School of Medicine, Nephrology and Internal Medicine, Bursa, Turkey

## Intr

Acute kidney injury (AKI) is characterized with decline in the glomerular filtration rate, elevation of serum blood urea nitrogen, creatinine, and other metabolic waste products [1]. AKI is considered to be associated with sepsis between 45 to 70% [2].

## Objectives

The aim of the study was to investigate the variabilities in vital signs, hemodynamic parameters and laboratory values after receiving renal replacement therapy (RRT) among AKI patients with or without sepsis in intensive care unit (ICU). Also we examined the two different renal injury staging systems in the patients.

## Methods

After obtaining approval from the Ethics Committee, patients who were admitted to ICU between 01.01.2010 - 30.06.2014 and requiring RRT in the management of AKI were enrolled. They were divided into septic shock group or nonseptic group. Glasgow coma score (GCS), vital signs, laboratory values, vasopressor or inotropic agents requirements at initiation, 12^th^ and 24^th^ hours of RRT were collected. APACHE II scores, 28^th^ and 90^th^ day mortality were also recorded. Staging of AKI was carried out based on KDIGO 2012 guidelines and RIFLE (Risk, Injury, Failure, Loss of kidney function and End-stage renal failure).

## Results

A total of 153 patients were included in the study. Ninety three patients had septic shock. APACHE II score and 28^th^ day mortality were significantly higher in Group Septic Shock (p < 0.001,p < 0.001 respectively). In subjects above 62 years of age found associated with higher 90^th^ day mortality (p = 0.002).

Both in two groups glomerular filtration rates, urea and creatinine levels improved after RRT initiation (p < 0.001). Also inotropic and vasopressor agent using has decreased significantly after RRT in both groups (p < 0.001).

Frequency of AKI were comparable with the KDIGO guideline and RIFLE classification (p = 0,5, p = 0,5 respectively).

## Conclusions

Mortality rate increases when AKI complicated with sepsis and higher age. The KDIGO staging for AKI is equivalent to the RIFLE classification in septic or non-septic critically ill patients.
